# Evaluation of Osteoconduction of Biphasic Calcium Phosphate Ceramic in the Calvaria of Rats: Microscopic and Histometric Analysis

**DOI:** 10.3390/jfb10010007

**Published:** 2019-01-17

**Authors:** Igor de Oliveira Puttini, Pier Paolo Poli, Carlo Maiorana, Igor Rodrigues de Vasconcelos, Luis Eduardo Schmidt, Luara Teixeira Colombo, Henrique Hadad, Gabriel Mulinari dos Santos, Paulo Sergio Perri de Carvalho, Francisley Ávila Souza

**Affiliations:** 1 Department of Surgery and Integrated Clinic, Araçatuba Dental of School, São Paulo State University Júlio de Mesquita Filho—UNESP, Araçatuba, SP 16 015 050, Brazil; igorputtini@gmail.com (I.d.O.P.); luara_colombo@hotmail.com (L.T.C.); henriquehadad@gmail.com (H.H.); gabriel_mulinari@hotmail.com (G.M.d.S.); psperri@foa.unesp.br (P.S.P.d.C.); 2 Implant Center for Edentulism and Jawbone Atrophies, Maxillofacial Surgery and Odontostomatology Unit, Fondazione IRCSS Cà Granda Maggiore Policlinico Hospital, University of Milan, 20122 Milan, Italy; pierpaolo.poli@unimi.it (P.P.P.); carlo.maiorana@unimi.it (C.M.); 3 Implant Dentistry Post-Graduation Program, São Leopoldo Mandic School of Dentistry and Research Center, Campinas, SP 13 045 755, Brazil; ivasco@hotmail.com (I.R.d.V.); luiz.eduardo.schmidt@gmail.com (L.E.S.)

**Keywords:** bone substitute, bone regeneration, biphasic calcium phosphate, ceramics

## Abstract

(1) Background: Evaluate the osteoconduction capability of a biphasic calcium phosphate (BCP) ceramic composed of hydroxyapatite and β-tricalcium phosphate 60%/40% in a rat model. (2) Methods: In the calvarial bone of 54 adult male rats, 7-mm diameter critical size defects were performed. The animals were randomly allocated to three experimental groups according to the type of material: blood clot (BCG), blood clot covered with a bovine-derived collagen membrane (MBCG), and BCP ceramic covered with a bovine-derived collagen membrane (BCPG). In each group, 6 animals were euthanatized at post-operative days 7, 30, and 60 for histological and histometric analysis. (3) Results: The qualitative analysis revealed the persistence of the collagen membrane at seven days, with no relevant newly bone formation in all groups. At 30 days, centripetal bone formation was observed residual particles of the biomaterial surrounded by fibroblasts noted in the BCPG. At 60 days, while BCG and MBCG showed a partial maturation with the central part of the defect populated by a fibrous connective tissue, in the BCPG the critical area was entirely occupied by newly formed bone. In the intra groups analysis was noted a significant increase in new bone formation during the experimental period (*p* < 0.05). At 60 days, BCPG showed a higher percentage area of new bone formation (*p* < 0.05). (4) Conclusion: BCP promoted a new bone formation by osteoconduction and might be considered a valid alternative in bone regeneration procedures.

## 1. Introduction

Replacement of missing teeth with osseointegrated dental implants is a well-established treatment option associated with high long-term survival rates [1]. The prognosis of implant-supported rehabilitations is promising for both total and partial edentulisms up to 20 years of clinical function [2,3]. Comparable outcomes have been reported for implants placed in native and in regenerated bone, emphasizing the importance of an adequate amount of hard tissue needed to obtain predictable results [4].

In this respect, a lack of bone is mainly due to the physiologic bone remodeling occurring in response to the loss of function as a consequence of tooth extraction. This may be exacerbated by other conditions, including dento-alveolar trauma, traumatic dental extractions, dental congenital absence, tumor resections, chronic/acute infections or the consequence of severe periodontal disease. The final result is a recipient site that is unsuitable to properly receive a dental implant, as an inadequate bone volume is a factor that negatively affects the primary stability and the long-term outcome [5].

The bone tissue has an intrinsic self-repairing potential that involves a tightly regulated sequential process, capable of restoring structure and functions [6]. However, the irreversible remodeling of the alveolar process after tooth loss leads to a decrease in the height and width of the residual ridge [7]. In these situations, bone grafting procedures are mandatory to recreate adequate volume and contour of the alveolar ridge in order to provide appropriate hygiene conditions, functions, and aesthetics of the implant-supported rehabilitation [8]. In this context, autogenous bone is bone graft gold standard combining osteoconductive, osteoinductive, and osteogenic characteristics with no immunological reactions [9]. However, few limitations can be identified, including an unpredictable resorption rate and the need for a second surgical site, therefore increasing the post-operative morbidity among all [10]. This forced the development of bone substitutes able to overcome the limitations associated with the of use autogenous bone grafts.

Due to their bioactive properties and chemical similarity to the mineral phase of bone, calcium phosphate-based biomaterials (CaP) are widely used for bone regeneration [11]. In particular, research on CaP has been directed toward the investigation of hydroxyapatite (HA) and β-tricalcium phosphate (β-TCP) in the form of biphasic calcium phosphate (BCP) [12]. BCP consists of two individual CaP phases: a more stable (HA) and a more soluble phase (β-TCP) in different proportions. By modifying the HA/β-TCP ratio and, thus, the solubility of ceramic, it is possible to influence the pattern of osteoclastic resorption [13]. This allows better control over the biodegradation and stability of the biomaterial while promoting bone ingrowth due to its osteoconductive property [12]. 

In view of the aforesaid characteristics, encouraging results emerged when BCPs were used alone [14,15] or as carriers [16,17] to promote and enhance bone regeneration in oral surgery. On the other hand, the porosity [18,19] and configurations [20,21] of BCPs are still source of investigations in view of their influence on the bioactive and bioabsorption properties. Furthermore, depending on the configuration, BCP might be susceptible to external compressive forces if not protected properly and thus may collapse in non-containing defects [22].

A new BCP biomaterial (Graftys BCP^®^, Latin American Solutions—LAS, Brazil) marketed in the forms of granules, sticks, cylinders, and wedges, has been developed as an alternative to autogenous bone grafts in case of bone regeneration procedures. Graftys BCP^®^ is a microporous, mesoporous, and macroporous two-phase calcium phosphate ceramic made of 60% HA and 40% β-TCP that resorbs and is gradually replaced by newly formed bone during the healing process. These characteristics facilitate long-term volume stability by decelerating the overall resorption capabilities, promoting a more stable and uniform bone growth.

In view of the aforesaid, the aim of the present experimental study was to evaluate the osteoconductive potential of a BCP ceramic bone substitute in critical size rat calvarial defects.

## 2. Materials and Methods

The present experimental study was performed according to the Ethical Principles for Animal Experimentation adopted by the Brazilian College of Animal Experimentation (COBEA), and was submitted to and approved by the Ethics Committee on Animal Experimentation of the School of Dentistry, Sao Paulo State University, Araçatuba, Brazil, under the Process FOA-01245-2012.

### 2.1. Experimental Design

In total, 54 adult male rats (Rattus norvegicus, Albinus, Wistar), weighing from 450 to 500 g, were used during the experimental procedures. The animals were provided by the animal facility of the same University (Araçatuba, SP, Brazil), where they were kept in individual cages in air-conditioned environment, and were fed with standard solid food and water ad libitum throughout the experiment. The animals were randomly divided into three groups according to the type of material grafted in the critical size defect that was created. In the clot group (BCG), the critical size defect was filled with blood clot; in the membrane clot group (MBCG), the critical size defect was filled with blood clot and covered with a type 1 bovine-derived collagen membrane (GenDerm^®^, Genius Biomaterials—Baumer SA, São Paulo, Brazil); in the biphasic calcium phosphate ceramic group (BCPG), the critical size defect was filled with BCP ceramic particles (Graftys BCP^®^, Latin American Solutions—LAS, São Paulo, SP, Brazil) covered with a bovine-derived cortical membrane.

### 2.2. Surgical Procedures

Before the surgical procedures, all acclimatized animals were fasted for 12 h. General anesthesia was induced through intramuscular administration of ketamine hydrochloride 1% (Vetaset^®^—Fort Dodge, Saúde Animal LTDA, Campinas, São Paulo, Brazil Farmacêuticos LTDA, Campinas, Brazil), at a dose of 10 mg/kg and xylazine hydrochloride 2% (Dopaser^®^—Laboratório Calier do Brasil Ltda, São Paulo, Brazil), at a dose of 5 mg/kg.

Manual shaving was then performed in the frontoparietal region. Subsequently, each animal was placed in a prone position in a sterile field, and a meticulous antisepsis of the surgical area was accomplished with topic polyvinylpyrrolidone solution (PVP aqueous 10%, with 1% active iodine, Riodeine^®^, Rioquímica, Sao Jose do Rio Preto, SP, Brazil).

The calvarial surgical access consisted of a median linear incision of 2 cm performed with a scalpel blade number 15C (Feather Industries Ltda, Tokyo, Japan) mounted on a number 3 scalpel handle (Hu-Friedy^®^, Chicago, IL, USA). The musculocutaneous flap was raised and retracted with surgical retractors to expose the parietal bone on both sides. A 7-mm trephine drill (Neodent^®^, Curitiba, Paraná, Brazil) mounted on a 20:1 reduction contra-angle (Kavo^®^ do Brasil, Joinvile, Brazil) connected to a straight surgical handpiece (BLM 600 plus, Driller^®^, Jaguaré, São Paulo, Brazil) at a speed of 1500 rpm was used to prepare a bicortical osteotomy in the middle region between the parietal bones. The osteotomized parietal bone was carefully removed to preserve the dura mater [23,24].

**Figure 1:**
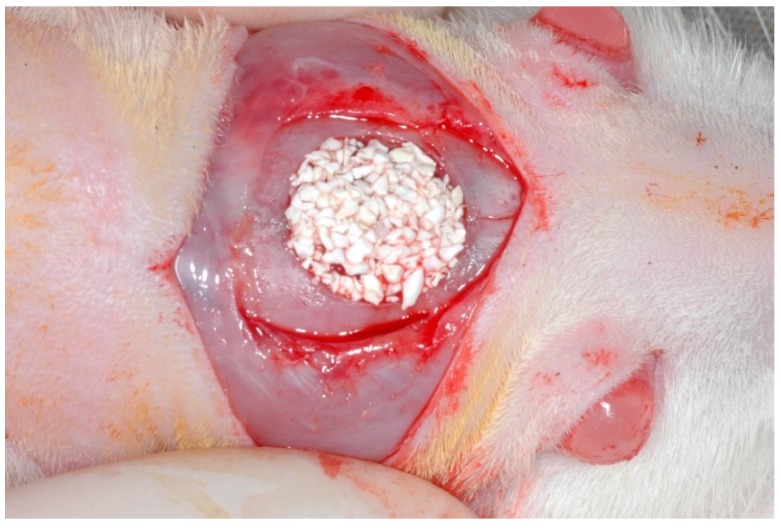
Biphasic calcium phosphate granules grafted in the critical size defect.

The critical size defect was then grafted according to the group allocation (Figure 1). In groups MBCG and BCPG, the collagen membrane was trimmed in a circular shape and laid over the defect to secure the graft. Finally, the musculocutaneous flap was repositioned and sutured with nylon 5–0 (ETHILON^®^, Ethicon, Johnson, São José dos Campos, Brazil) simple interrupted stitches.

Euthanasia and specimen retrieval were performed on post-operative days 7, 30, and 60, upon six animals per group for each time period. The animals were sacrificed with an overdose of anesthetic.

### 2.3. Sample Preparation

Osteotomies were performed on each calvaria to obtain bone samples with at least 3 cm of margins circumferentially around the grafted area. The pieces were immersion-fixed in 10% neutral buffered formalin (Analíticos^®^ Reagents, Dental-Hospital Dynamics Ltd., Catanduva, SP, Brazil) and decalcified in 20% ethylenediaminetetraacetic acid (EDTA, Merck Millipore, Barueri, SP, Brazil) dissolved in Milli-Q water (Merck Millipore, Barueri, SP, Brazil), changed weekly over a period of six weeks at room temperature. The samples were then dehydrated in an ascending series of ethanol concentrations (70, 90, 95, and absolute ethanol), with the solution being replaced every 1 h, in an orbital shaker (KLine CT—150^®^, Cientec—Laboratory equipment, Piracicaba, SP, Brazil). The samples were successively cleared in xylol, paraffin-embedded and prepared using a precision saw to obtain 6-μm thick sections. The sections were mounted onto slides and stained with hematoxylin eosin (HE Merck and Co., Inc., Rahway, NJ, USA). The slides were finally coded to perform a blind histometric analysis.

### 2.4. Histological and Histometric Analysis

A single investigator performed the histological analysis, blinded with respect to the group allocation. A qualitative histological description was performed for all specimens. The histometric evaluation were performed using ImageJ^®^ (U.S. National Institutes of Health, Bethesda, MD, USA), version 3.1, and the area of newly formed bone was expressed in micrometers and converted in percentage as the ratio of newly formed bone area/total area × 100. Five sections from each sample were prepared in a plane parallel to the sagittal suture and through the center of the augmented area and stained with HE. At the surface of the calvaria bone, we determined the newly formed bone inside the calvarial defect without difference between the groups. The slices were scanned using an optical microscope (Leica DMLB, Heerbrugg, Switzerland) paired with an image capture camera (Leica DC 300F Microsystems Ltd, Heerbrugg, Switzerland) and connected to a microcomputer Intel core i5 (Dell Computadores do Brasil Ltda, Hortolandia, SP, Brazil). The slides were photomicrographs magnified from the originals by 4× and 40×. The scanned images were recorded in JPEG-format files and analyzed using the “hands free” tools in the Image J^®^ (U.S. National Institutes of Health, Bethesda, MD, USA), and then the area of newly formed bone was selected inside all area of the calvarial defect, even in the area of connective tissue or biomaterial, and blood clot.

### 2.5. Statistical Analysis

The VPR independent statistician performed the statistical analysis. Initially, data were submitted to normality Shapiro-Wilk test, which showed normal distribution (*p* < 0.05). Hence, an ANOVA test and, when necessary, post hoc Tukey’s test was applied to compare the experimental times (7, 30, and 60 days) and types of graft within groups (BCG, MBCG and BCPG). A linear regression model was used to assess the time effect (in days) with respect to the daily percentage increase of new bone formation in each group. A significance level of 0.05 was adopted for all tests.

## 3. Results

### 3.1. Qualitative Analysis

**Figure 2:**
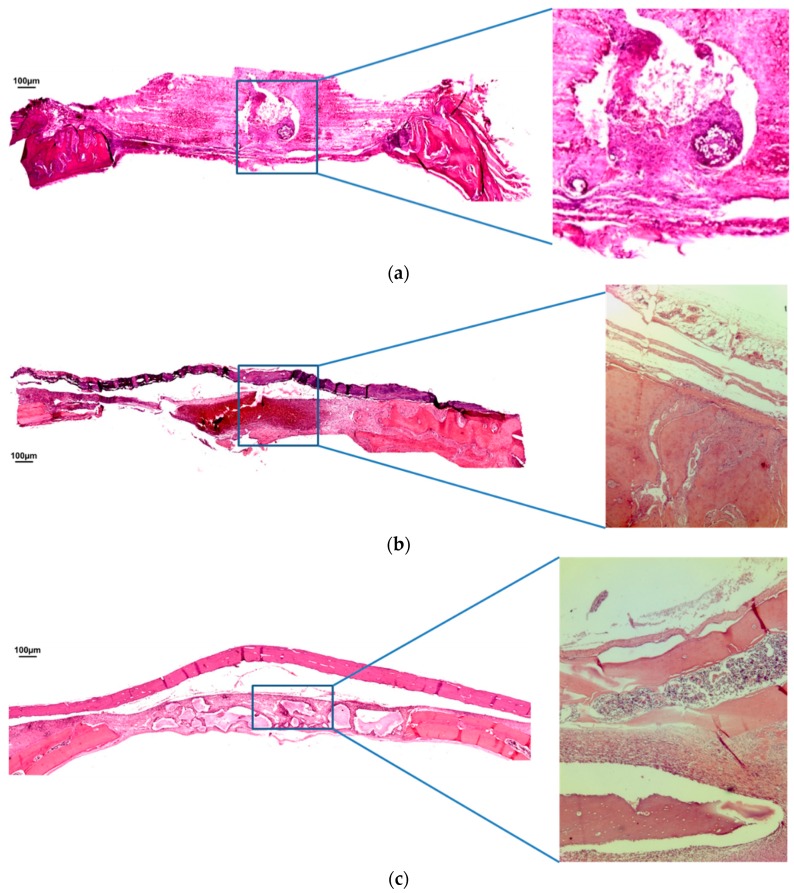
(**a**) Hematoxylin and eosin staining at seven days of BCG. (**b**) Hematoxylin and eosin staining at seven days of MBCG. (**c**) Hematoxylin and eosin staining at seven days of BCPG. (Magnification: 4×; 40×).

At seven days in BCG, almost no new bone formation could be observed (Figure 2a). The blood clot was arranged within the osteotomy lines with no significant inflammatory infiltration. In both MBCG (Figure 2b) and BCPG (Figure 2c), the bovine cortical membrane could be identified over the defect area. The blood clot was organized within the region of interest and was associated with a mild inflammatory infiltrate. It was not possible to detect relevant newly bone formation in the critical size defect. In BCPG, the biomaterial particles were encapsulated into a fibroblast-like tissue.

**Figure 3:**
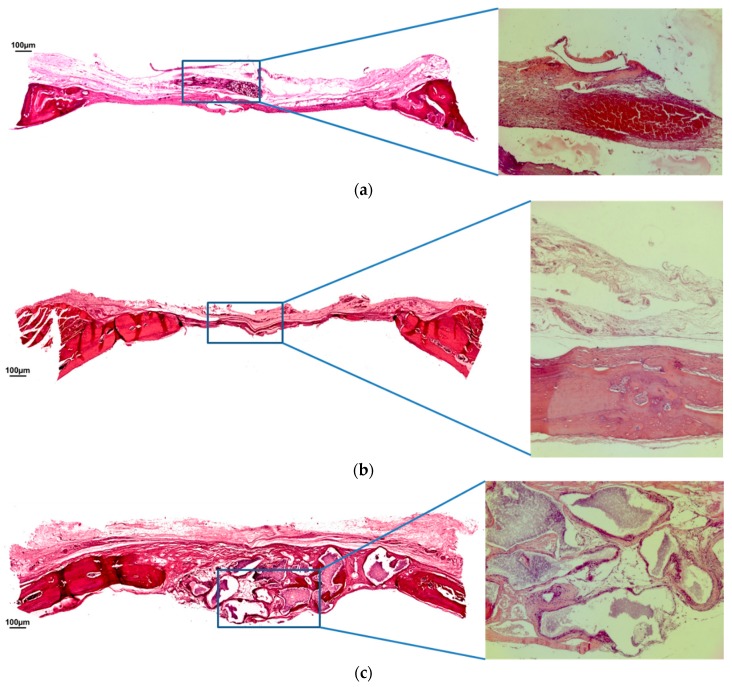
(**a**) Hematoxylin and eosin staining at 30 days of BCG. (**b**) Hematoxylin and eosin staining at 30 days of MBCG. (**c**) Hematoxylin and eosin staining at 30 days of BCPG. (Magnification: 4×; 40×).

After 30 days, the BCG exhibited a significant newly bone formation originating from the margins of the defect and directed toward the inner portion (Figure 3a). A loose immature connective tissue was observed between the growing bone trabeculae. In MBCG and BCPG, the bioabsorbable membrane was completely resorbed. In MBCG, a centripetal bone formation could be observed, however the central regions were still characterized by a loose immature connective tissue (Figure 3b). In BCPG, similarly to the other groups, newly bone formation occurred primarily in the external area. The more central portion was characterized by residual particles of the biomaterial surrounded by fibroblasts. In this central area, islands of newly formed bone were clearly visible, highlighting the substitution process of the biomaterial resulting in apposition of newly formed bone (Figure 3c).

**Figure 4:**
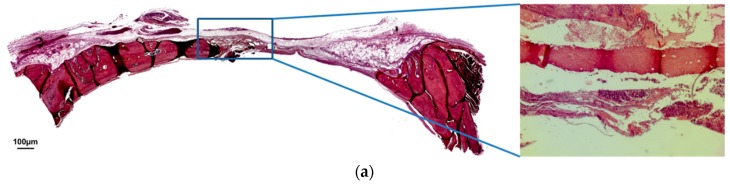

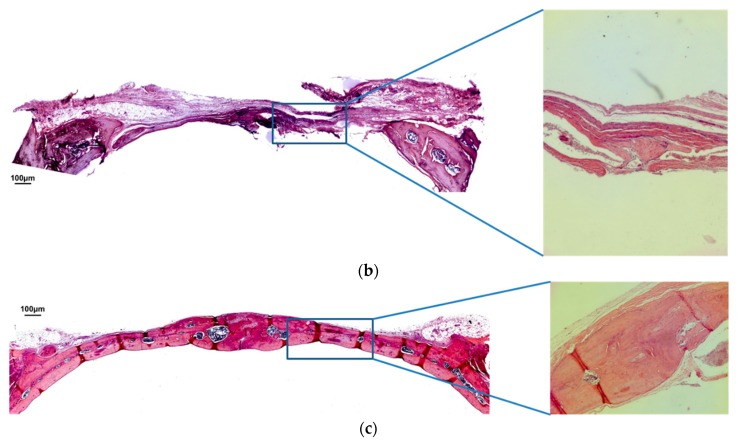
(**a**) Hematoxylin and eosin staining at 60 days of BCG. (**b**) Hematoxylin and eosin staining at 60 days of MBCG. (**c**) Hematoxylin and eosin staining at 60 days of BCPG. (Magnification: 4×; 40×).

At 60 days, the BCG showed a partial maturation, with roughly 50% of the defect completely ossified. The rest of the defect was populated by a fibrous connective tissue (Figure 4a). The MBCG exhibited a similar healing pattern, with roughly 40% of the defect completely ossified (Figure 4b). On the other hand, in the BCPG it was possible to observe a complete maturation of the defect, with the critical area entirely occupied by newly formed bone (Figure 4c).

### 3.2. Quantitative Analysis

The comparative analysis of the percentage of newly bone formation was expressed in Table 1. At 7 days the area of newly bone formation in the BCPG group was higher when compared with the BCG group (*p* = 0.013). The MBCG have not presented statistically significant differences when compared to the other two groups. At 30 days the area of new bone formation has not showed differences statistically significant between groups (*p* = 0.065). At 60 days BCPG showed a higher area of new bone formation when compared to MBCG and BCG (*p* < 0.001). The intra-group analysis showed a statistically significant increase in new bone formation (*p* < 0.05) at 60 days. The influence of the duration of the experimentation in regards to the bone neoformation rate in each group was reported in Table 2. The highest daily increase of bone formation was estimated in BCPG, with a daily rate of 1.35% (R^2^ = 0.72%; *p* < 0.001), followed by the MBCG, with a daily rate of 0.24% (R^2^ = 0.63; *p* < 0.001), and BCG with a daily rate of 0.18% (R^2^ = 0.55; *p* < 0.001). The highest daily increase of bone formation was described in Table 2.

## 4. Discussion

The present experimental study reports on the successful application of a BCP biomaterial in critical size defects to investigate the osteoconductive potential of HA/β-TCP association. Biphasic HA/β-TCP is a bone substitute produced by a single process to prevent clustering and to establish a homogeneous molecule. Its specific 60%/40% HA/β-TCP ratio gives it two phases of activity. 

The association of β-TCP/HA provided a good level of osteoconduction favoring and facilitating the bone formation process. In all groups, a trend towards an increasing amount of newly formed bone was observed within the study periods. It is worthy of note that the defects filled with the BCP biomaterial reached the maximum of bone formation, closing the defect created in all animals within 60 days. The quantitative analysis is corroborated by the histological findings, showing a complete maturation of the defect, with the critical area entirely occupied by newly formed bone, in the BCPG group. Although the degree of reabsorption of biomaterial granules was not measured in this study, but it is possible to compare the degree of resorption of granules between the periods (7, 30, 60 days) of each group through the histological images.

An important aspect is related to the HA/β-TCP ratio, which might influence the resorption activity of osteoclasts by modifying the solubility of ceramic. The resorption and precipitation processes are regarded as major factors implicated in their bioactive properties. Resorption activity was observed on pure β-TCP and BCP with 25% HA and 75% β-TCP. Conversely, osteoclasts did not resorb BCP with a 75%/25% HA/β-TCP ratio or pure HA [25]. Another combination of 60%/40% HA/β-TCP has been tried clinically in humans with successful results [26].

Interestingly, scaffolds presenting a HA/β-TCP weight ratio of 60%/40% have shown beneficial effects for cells growth, as a confirmation of their biocompatibility, and thus might be considered as good candidates for bone tissue engineering applications [27]. Surprisingly, BCP scaffold composed of 60%/40% HA/β-TCP ratio provided minimal osteoinduction on ectopically-formed bone in a mouse model [28,29]. Accordingly, synthetically fabricated ceramics showed the capability of forming ectopic bone formation even in large animal models, with a healing pattern as rapid as the autogenous bone [30]. These finding seems to support positively our results, explaining the potential of BCP with a 60%/40% HA/β-TCP ratio to promote and assist new bone formation.

When transferred to the maxillary sinus compartment, BCP in the form of 60%/40% HA/β-TCP used as a bone substitute, exhibited biocompatibility and osteoconductive properties, promoting new bone formation after sinus elevation surgeries. It is noteworthy that the amount of new bone formation was significantly higher in the autogenous group compared to the BCP group [14]. Our results are more consistent with a recent animal study in which mandibular critical size defects in rabbit were graft with porous BCP or autogenous bone. In this model, neither statistically significant difference was found in newly formed bone between BCP and autograft groups at 4, 8, and 12 weeks [31].

Microscopic structure of HA/β-TCP offers granules with a porosity of 90%, and interconnected pores ranging from 100 to 500 μm in diameter to support cellular penetration. Actually, the graft material used in the present study presented 67% porosity, composed of 84% microporosity (<10 μm), 8% mesoporosity (10–100 μm), and 8% macroporosity (>100–150 μm). Interestingly, in evaluating the impact of total porosity of BCP on cell microenvironment, a similar porosity of 65% displayed a great odontoblastic differentiation strongly suggesting it can support human dental pulp cells differentiation for dentin tissue regeneration [32]. A part from the global porosity, the pore size might have played a pivotal role in governing the dynamic processes of vascularization and osseointegration of the bone substitute. It is likely that the microporosity was able to proportionate the flux of ions and organic fluids within the graft, and the mesoporosity allowed cellular mobility and the physiological cell signaling occurring between macropores. It has been demonstrated that micropore-induced capillarity is responsible for the enhancement in bone distribution [33]. A homogeneous bone distribution reduces the occurrence of empty spaces that jeopardize the mechanical strength of the grafted scaffold. In addition, functional vascularization throughout the scaffold decreases the risk for tissue necrosis at the center of the defect. These findings were observed in the present study, in which a homogeneous bone distribution was noted in the BCPG at the end of the study period. The macroporosity permitted a total colonization of the bone substitute by osteoprogenitors cells to promote new bone formation. This was supported by the fact that ceramics whose pore sizes exceed 140 μm showed a shorter onset of blood vessels formation, a higher functional capillary density, and a higher volume of newly formed bone with respect to lower pore sizes [34].

A particulated graft serves as a scaffold that promotes a tridimensional matrix, which will stabilize and maintain the organization of the blood clot. It also allows and supports cell migration and angiogenesis, resulting in new bone formation during repair. On the other hand, compressive forces by the surrounding soft tissues might dislodge the biomaterial from the defect, hampering the healing process. For this reason, the placement of an occlusive membrane has been introduced to prevent connective tissue cells from colonizing the defect and at the same time provide enough space to allow for bone regeneration of the entire defect volume preventing micromovements and compression [35]. The presence of the bioabsorbable membrane associated with biomaterial acts as fundamental role in osteoconduction, and serves as a barrier since it prevents the biomaterial from spreading out of the critical defect. This might explain the higher newly bone formation observed in the groups covered by the membrane with respect to the control group observed in the present study. The membrane allowed osteoblasts to enter and bone formation to proceed undisturbed and acted as a barrier to prevent fibroblasts from growing into the bony defect, supported by the underlying the particulated graft. Another similarity was observed evaluating the direct relationship between graft maturation time and vital bone formation. This trend of increased vital bone volume with increased healing time was also observed in the present study.

## 5. Conclusions

In conclusion, the results suggested that BCP composed of 60%/40% HA/β-TCP promoted a new bone formation by osteoconduction and might be considered a valid alternative in bone regeneration procedures. Further in vivo studies are needed to evaluate the optimal HA/β-TCP ratio and the ideal porosity of BCP-based bone substitutes.

## Figures and Tables

**Table 1:** Mean, standard deviations, and comparative analysis of the newly bone formation percentage with respect to the groups and experimental periods. Data are expressed as mean ± standard deviation (SD).

Experimental Period	Control Group (BCG)	Blood Clot + Membrane (MBCG)	BCP + Membrane (BCPG)	*p* Value
	Mean ± SD	Mean ± SD	Mean ± SD	
7 days	0.76 ± 0.70 ^Aa^	1.73 ± 1.13 ^ABa^	4.52 ± 3.16 ^Ba^	0.013 *
30 days	4.45 ± 3.92 ^Aa^	6.04 ± 1.16 ^Aa^	8.22 ± 1.23 ^Aa^	0.065
60 days	10.66 ± 5.57 ^Ab^	14.51 ± 7.00 ^Ab^	74.22 ± 25.39 ^Bb^	<0.001 *
*p* value	0.002 *	<0.001 *	<0.001 *	

* Statistically significant value (*p* < 0.05 by ANOVA test). A and B: shows the difference between the groups in the same period (*p* < 0.05 by Tukey test); a and b: shows the difference between the period in the same group (*p* < 0.05 by Tukey test).

**Table 2:** Effect of the time period (in days) estimated with the linear regression model with respect to the percentage of new bone increase.

Groups	R²	β	*p* Value
Control group (BCG)	0.55	0.18	<0.001 *
Blood clot + membrane (MBCG)	0.64	0.24	<0.001 *
BCP + membrane (BCPG)	0.72	1.35	<0.001 *

R² = Coefficient of determination; β = Standardized coefficient; * Statistically significant value.

## List of Abbreviations

BCP	Biphasic calcium phosphate
BCG	Blood clot group
MBCG	Membrane blood clot group
BCPG	Biphasic calcium phosphate group
CaP	Calcium phosphate-based biomaterials
HA	Hydroxyapatite
β-TCP	Β-tricalcium phosphate
HA/β-TCP	Composite of hydroxyapatite and β-tricalcium phosphate
COBEA	Brazilian College of Animal Experimentation
FOA	Araçatuba Dental of School
Milli-Q water	Deionized water
μm	Micrometers
%	Percentage
R²	Coefficient of determination
β	Standardized coefficient
